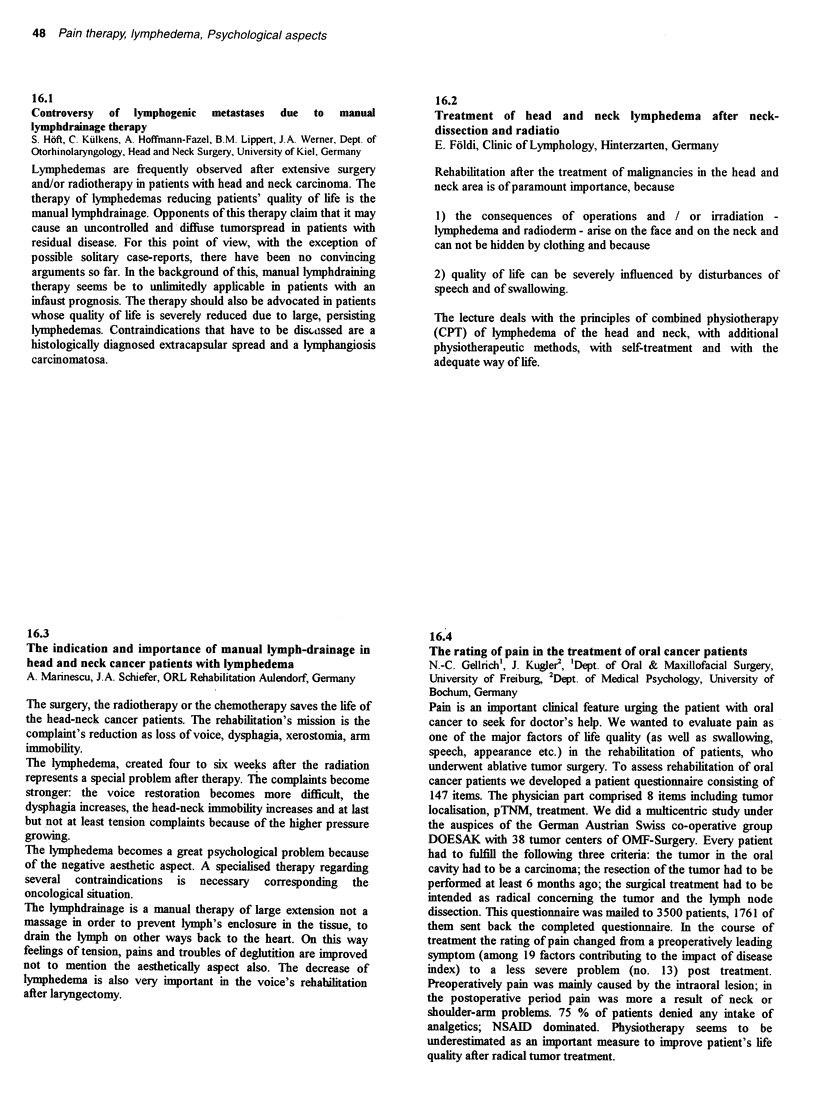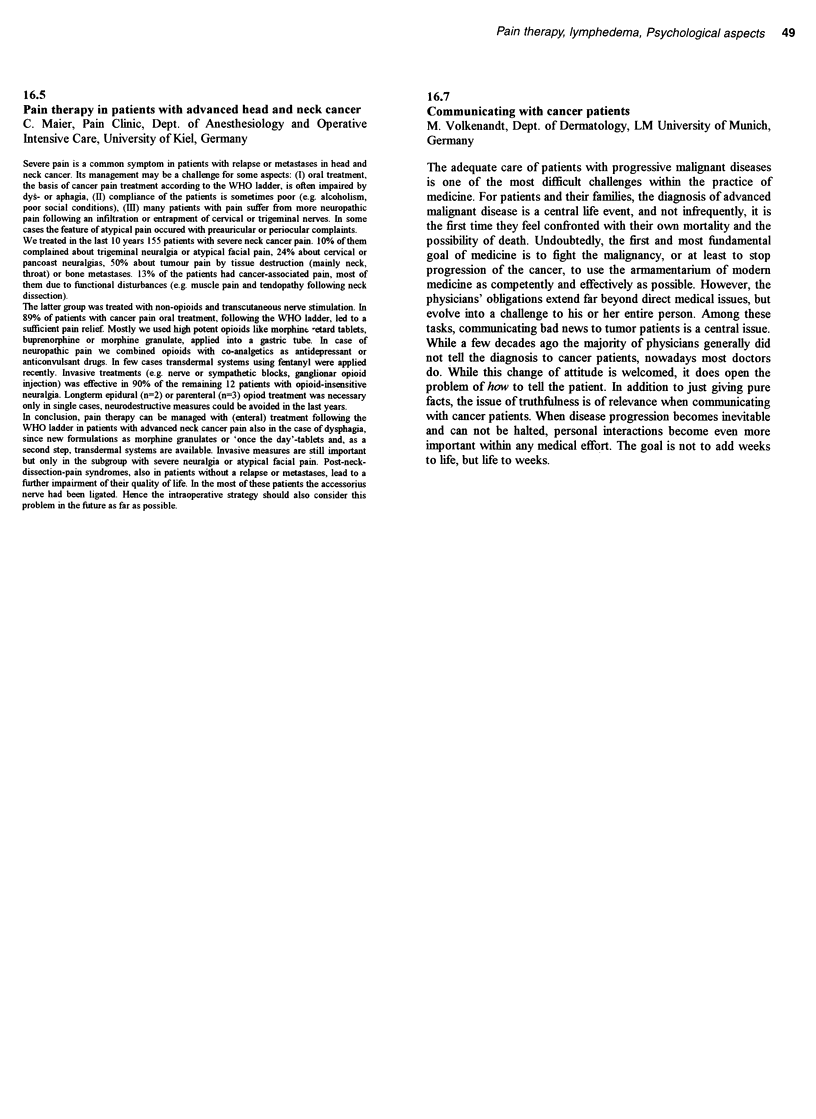# Pain therapy, Treatment of Lymphoedema, Psychological aspects of cancer Patients

**Published:** 1998

**Authors:** 


### 16.1
### Controversy of lymphogenic metastases due to manual lymphdrainage therapy

S. Höft, C. Külkens, A. Hoffmann-Fazel, B.M. Lippert, J.A. Werner, Dept. of Otorhinolaryngology, Head and Neck Surgery, University of Kiel, Germany

Lymphedemas are frequently observed after extensive surgery and/or radiotherapy in patients with head and neck carcinoma. The therapy of lymphedemas reducing patients' quality of life is the manual lymphdrainage. Opponents of this therapy claim that it may cause an uncontrolled and diffuse tumorspread in patients with residual disease. For this point of view, with the exception of possible solitary case-reports, there have been no convincing arguments so far. In the background of this, manual lymphdraining therapy seems be to unlimitedly applicable in patients with an infaust prognosis. The therapy should also be advocated in patients whose quality of life is severely reduced due to large, persisting lymphedemas. Contraindications that have to be discussed are a histologically diagnosed extracapsular spread and a lymphangiosis carcinomatosa.

### 16.2
### Treatment of head and neck lymphedema after neck-dissection and radiatio

E. Földi, Clinic of Lymphology, Hinterzarten, Germany

Rehabilitation after the treatment of malignancies in the head and neck area is of paramount importance, because

1) the consequences of operations and / or irradiation - lymphedema and radioderm - arise on the face and on the neck and can not be hidden by clothing and because

2) quality of life can be severely influenced by disturbances of speech and of swallowing.

The lecture deals with the principles of combined physiotherapy (CPT) of lymphedema of the head and neck, with additional physiotherapeutic methods, with self-treatment and with the adequate way of life.

### 16.3
### The indication and importance of manual lymph-drainage in head and neck cancer patients with lymphedema

A. Marinescu, J.A. Schiefer, ORL Rehabilitation Aulendorf, Germany

The surgery, the radiotherapy or the chemotherapy saves the life of the head-neck cancer patients. The rehabilitation's mission is the complaint's reduction as loss of voice, dysphagia, xerostomia, arm immobility.

The lymphedema, created four to six weeks after the radiation represents a special problem after therapy. The complaints become stronger: the voice restoration becomes more difficult, the dysphagia increases, the head-neck immobility increases and at last but not at least tension complaints because of the higher pressure growing.

The lymphedema becomes a great psychological problem because of the negative aesthetic aspect. A specialised therapy regarding several contraindications is necessary corresponding the oncological situation.

The lymphdrainage is a manual therapy of large extension not a massage in order to prevent lymph's enclosure in the tissue, to drain the lymph on other ways back to the heart. On this way feelings of tension, pains and troubles of deglutition are improved not to mention the aesthetically aspect also. The decrease of lymphedema is also very important in the voice's rehabilitation after laryngectomy.

### 16.4
### The rating of pain in the treatment of oral cancer patients

N.-C. Gellrich[1], J. Kugler[2], [1]Dept. of Oral & Maxillofacial Surgery, University of Freiburg, [2]Dept. of Medical Psychology, University of Bochum, Germany

Pain is an important clinical feature urging the patient with oral cancer to seek for doctor's help. We wanted to evaluate pain as one of the major factors of life quality (as well as swallowing, speech, appearance etc.) in the rehabilitation of patients, who underwent ablative tumor surgery. To assess rehabilitation of oral cancer patients we developed a patient questionnaire consisting of 147 items. The physician part comprised 8 items including tumor localisation, pTNM, treatment. We did a multicentric study under the auspices of the German Austrian Swiss co-operative group DOESAK with 38 tumor centers of OMF-Surgery. Every patient had to fulfill the following three criteria: the tumor in the oral cavity had to be a carcinoma; the resection of the tumor had to be performed at least 6 months ago; the surgical treatment had to be intended as radical concerning the tumor and the lymph node dissection. This questionnaire was mailed to 3500 patients, 1761 of them sent back the completed questionnaire. In the course of treatment the rating of pain changed from a preoperatively leading symptom (among 19 factors contributing to the impact of disease index) to a less severe problem (no. 13) post treatment. Preoperatively pain was mainly caused by the intraoral lesion; in the postoperative period pain was more a result of neck or shoulder-arm problems. 75 % of patients denied any intake of analgetics; NSAID dominated. Physiotherapy seems to be underestimated as an important measure to improve patient's life quality after radical tumor treatment.

### 16.5
### Pain therapy in patients with advanced head and neck cancer

C. Maier, Pain Clinic, Dept. of Anesthesiology and Operative Intensive Care, University of Kiel, Germany

Severe pain is a common symptom in patients with relapse or metastases in head and neck cancer. Its management may be a challenge for some aspects: (I) oral treatment, the basis of cancer pain treatment according to the WHO ladder, is often impaired by dyš- or aphagia, (II) compliance of the patients is sometimes poor (e.g. alcoholism, poor social conditions), (III) many patients with pain suffer from more neuropathic pain following an infiltration or entrapment of cervical or trigeminal nerves. In some cases the feature of atypical pain occured with preauricular or periocular complaints.
We treated in the last 10 years 155 patients with severe neck cancer pain. 10% of them complained about trigeminal neuralgia or atypical facial pain, 24% about cervical or pancoast neuralgias, 50% about tumour pain by tissue destruction (mainly neck, throat) or bone metastases. 13% of the patients had cancer-associated pain, most of them due to functional disturbances (e.g. muscle pain and tendopathy following neck dissection).
The latter group was treated with non-opioids and transcutaneous nerve stimulation. In 89% of patients with cancer pain oral treatment, following the WHO ladder, led to a sufficient pain relief. Mostly we used high potent opioids like morphine retard tablets, buprenorphine or morphine granulate, applied into a gastric tube. In case of neuropathic pain we combined opioids with co-analgetics as antidepressant or anticonvulsant drugs. In few cases transdermal systems using fentanyl were applied recently. Invasive treatments (e.g. nerve or sympathetic blocks, ganglionar opioid injection) was effective in 90% of the remaining 12 patients with opioid-insensitive neuralgia. Longterm epidural (n=2) or parenteral (n=3) opiod treatment was necessary only in single cases, neurodestructive measures could be avoided in the last years.
In conclusion, pain therapy can be managed with (enteral) treatment following the WHO ladder in patients with advanced neck cancer pain also in the case of dysphagia, since new formulations as morphine granulates or 'once the day'-tablets and, as a second step, transdermal systems are available. Invasive measures are still important but only in the subgroup with severe neuralgia or atypical facial pain. Post-neck-dissection-pain syndromes, also in patients without a relapse or metastases, lead to a further impairment of their quality of life. In the most of these patients the accessorius nerve had been ligated. Hence the intraoperative strategy should also consider this problem in the future as far as possible.

### 16.7
### Communicating with cancer patients

M. Volkenandt, Dept. of Dermatology, LM University of Munich, Germany

The adequate care of patients with progressive malignant diseases is one of the most difficult challenges within the practice of medicine. For patients and their families, the diagnosis of advanced malignant disease is a central life event, and not infrequently, it is the first time they feel confronted with their own mortality and the possibility of death. Undoubtedly, the first and most fundamental goal of medicine is to fight the malignancy, or at least to stop progression of the cancer, to use the armamentarium of modern medicine as competently and effectively as possible. However, the physicians' obligations extend far beyond direct medical issues, but evolve into a challenge to his or her entire person. Among these tasks, communicating bad news to tumor patients is a central issue. While a few decades ago the majority of physicians generally did not tell the diagnosis to cancer patients, nowadays most doctors do. While this change of attitude is welcomed, it does open the problem of *how* to tell the patient. In addition to just giving pure facts, the issue of truthfulness is of relevance when communicating with cancer patients. When disease progression becomes inevitable and can not be halted, personal interactions become even more important within any medical effort. The goal is not to add weeks to life, but life to weeks.